# Two-Point Localization Algorithm of a Magnetic Target Based on Tensor Geometric Invariant

**DOI:** 10.3390/s24072224

**Published:** 2024-03-30

**Authors:** Cheng Chi, Dan Wang, Ronghua Tao, Jianwei Li, Ye Wang, Zhentao Yu, Lu Yu

**Affiliations:** Institute of Remote Sensing, Navy Submarine Academy, Qingdao 266000, China

**Keywords:** magnetic gradient tensor, Levenberg–Marquardt algorithm, geometric invariant, magnetic target localization

## Abstract

Currently, magnetic gradient tensor-based localization methods face challenges such as significant errors in geomagnetic field estimation, susceptibility to local optima in optimization algorithms, and inefficient performance. In addressing these issues, this article propose a two-point localization method under the constraint of overlaying geometric invariants. This method initially establishes the relationship between the target position and the magnetic gradient tensor by substituting an intermediate variable for the magnetic moment. Exploiting the property of the eigenvector corresponding to the minimum absolute eigenvalue being perpendicular to the target position vector, this constraint is superimposed to formulate a nonlinear system of equations of the target’s position. In the process of determining the target position, the Nara method is employed for obtaining the initial values, followed by the utilization of the Levenberg–Marquardt algorithm to derive a precise solution. Experimental validation through both simulations and experiments confirms the effectiveness of the proposed method. The results demonstrate its capability to overcome the challenges faced by a single-point localization method in the presence of some errors in geomagnetic field estimation. In comparison to traditional two-point localization methods, the proposed method exhibits the highest precision. The localization outcomes under different noise conditions underscore the robust noise resistance and resilience of the proposed method.

## 1. Introduction

Under the influence of the Earth’s magnetic field, ferromagnetic targets inevitably undergo magnetization, leading to alterations in the distribution of the ambient magnetic field and the generation of magnetic anomalies [1]. These anomaly signals serve as crucial sources for detecting and locating magnetic targets [2]. Magnetic detection, known for its advantages of passive and high-precision detection, has gained widespread attention. Currently, it finds applications in various fields such as underwater intrusion detection [3], UXO detection, iron ore exploration [4], endoscopic capsule detection, and more.

In comparison to scalar [5,6] and vector [7,8,9] magnetic measurements, magnetic gradient tensors provide rich information about the source field and exhibit the advantage of being less influenced by the Earth’s magnetic field. Consequently, they have garnered significant attention in recent years.

The measurement of magnetic gradient tensors, providing information on nine components containing source field information, has made methods utilizing this information for target localization a research hotspot. These localization methods can essentially be categorized into two types: single-point and multi-point methods. A prominent algorithm in single-point localization is the method proposed by Nara et al. [10], from the University of Tokyo, which utilizes a target’s magnetic gradient tensor and vector field information to locate magnetic dipoles. This method calculates the target’s position information through a closed-form localization formula, making the computation straightforward. However, it relies on the target’s vector field information, which is challenging to separate from the geomagnetic background field, introducing estimation errors. Nara [11] later addressed the singularity issue in this method during localization analysis. In response to this problem, Yin Gang [12] proposed a corresponding solution. Nonetheless, these methods still face challenges associated with errors in estimating the geomagnetic field.

To overcome issues in single-point localization methods, several scholars proposed corresponding improvement methods [13,14,15]. Typically, the approach involves not directly utilizing the magnetic field vector term from the original Nara algorithm. For instance, in reference [16,17], methods using higher-order tensor information for target localization were proposed. Due to the small magnitude of the magnetic gradient tensor after differencing and its susceptibility to measurement noise, these methods have high demands on the dynamic noise of the measuring instrument. In reference [18], the characteristic of approximately equal geomagnetic field values at two closely spaced measurement points is exploited. An equation system of the target’s position is established for localization. However, this method requires a specific magnetic gradient tensor measurement structure to simultaneously measure information from two points, making it structurally complex. Reference [19] replaces the target’s magnetic moment with the target’s vector magnetic field values and constructs a nonlinear objective function of the target position parameters. A genetic algorithm is then employed to solve the function with these parameters. However, the above optimization algorithm only utilizes the relationship between the positions of two measurement points and the measured values of the magnetic gradient tensor. The success rate of optimization is low, and it is greatly affected by the initial values. Liu Huan [20] introduced a constraint term, magnetic moment constraints, to the aforementioned optimization algorithm, reducing localization errors. However, the issue of easily falling into a local optimum still persists.

The property of tensor invariants, remaining unchanged with variations in the carrier’s orientation, has garnered significant attention. Consequently, the method of utilizing magnetic gradient tensor invariants for localization has been extensively researched. Wiegert et al. [21], from the U.S. Naval Surface Warfare Center, introduced a method (STAR) utilizing magnetic gradient tensor invariants for localization. Subsequently, several scholars [22,23,24] conducted research on the properties satisfied by these invariants and proposed methods for localization using them. The method of using invariants for positioning is essentially a multi-point positioning method. However, achieving real-time localization with invariants requires a complex magnetic gradient tensor measurement system structure, involving a considerable number of magnetic fluxgate sensors. This complexity results in a higher number of error parameters to be corrected [25,26,27,28], posing challenges in practical applications.

In summary, the current single-point magnetic gradient tensor localization methods face challenges from the errors in estimating the geomagnetic field. Simultaneously, multi-point localization methods encounter issues of low optimization efficiency and a susceptibility to falling into local optima. To address these challenges, a two-point magnetic gradient tensor localization method is proposed, incorporating constraints from tensor geometric invariants. This method utilizes magnetic gradient tensor measurement data from two points, superimposes constraints from tensor geometric invariants, constructs a nonlinear objective function regarding the target’s position coordinates, and employs the Nara localization method to compute the initial target position. This initial value is then input into the Levenberg–Marquardt algorithm to obtain an accurate solution for the target’s position parameters. Finally, the effectiveness of the positioning method proposed in this paper is validated through simulation experiments using three measurement paths and experimental tests using one measurement path. The experimental results indicate that the proposed method can achieve the precise positioning of magnetic targets.

## 2. The Proposed Two-Point Localization Algorithm

### 2.1. Principle of Magnetic Gradient Tensor Localization

When the distance between the magnetic target and the magnetic sensor exceeds 2.5 times the characteristic length of the magnetic target, the magnetic target can be considered as a magnetic dipole [2]. The magnetic dipole model is represented as follows:

(1) $$B=\frac{\mu }{4\pi }\left[\frac{3(m\cdot r)r}{r^{5}}-\frac{m}{r^{3}}\right],$$

where ***m*** represents the magnetic moment of the magnetic target, ***r*** is the distance vector from the magnetic target to the magnetic sensor, and μ is the magnetic permeability constant in the air, with a magnitude of $4\pi \times 10^{-7}$ Tm/A.

The magnetic gradient tensor is defined as the rate of change in the magnetic field’s three components along the three coordinate axes. The expression is as follows:

(2) $$G=\nabla B=\left[\begin{array}{ccc} \partial B_{x}/\partial x & \partial B_{x}/\partial y & \partial B_{x}/\partial z\\ \partial B_{y}/\partial x & \partial B_{y}/\partial y & \partial B_{y}/\partial z\\ \partial B_{z}/\partial x & \partial B_{z}/\partial y & \partial B_{z}/\partial z \end{array}\right]=\left[\begin{array}{ccc} B_{xx} & B_{xy} & B_{xz}\\ B_{yx} & B_{yy} & B_{yz}\\ B_{zx} & B_{zy} & B_{zz} \end{array}\right].$$

The magnetic gradient tensor consists of nine components, and the expressions for each component are as follows:

(3) $$B_{ij}=\frac{\partial B_{i}}{\partial r_{j}}=\frac{3\mu }{4\pi }\left[\frac{(m\cdot r)\delta_{ij}+r_{i}m_{j}+r_{j}m_{i}}{r^{5}}-\frac{5r_{i}r_{j}(m\cdot r)}{r^{7}}\right],$$

where i and j represent the three components in the Cartesian coordinate system. Because the gradient of the geomagnetic field is less than or equal to 0.02 nT/m, using the magnetic gradient tensor for localization can eliminate interference from the geomagnetic field. In regions without conduction currents, the divergence and curl of the magnetic flux density are both zero. Therefore, the tensor is traceless and symmetric. From Equation (3), it can be deduced that, among the nine components of the magnetic gradient tensor, only five are independent. By measuring these five components, the magnetic gradient tensor at that point can be determined. The expressions for these five independent components are as follows:

(4) $$\left[\begin{array}{c} B_{xx}\\ B_{xy}\\ B_{xz}\\ B_{yy}\\ B_{yz} \end{array}\right]=\frac{\mu }{4\pi r^{7}}\left[\begin{array}{ccc} 9xr^{2}-15x^{3} & 3yr^{2}-15x^{2}y & 3zr^{2}-15x^{2}z\\ 3yr^{2}-15x^{2}y & 3xr^{2}-15xy^{2} & -15xyz\\ 3zr^{2}-15x^{2}z & -15xyz & 3xr^{2}-15xz^{2}\\ 3xr^{2}-15xy^{2} & 9yr^{2}-15y^{3} & 3zr^{2}-15y^{2}z\\ -15xyz & 3zr^{2}-15y^{2}z & 3yr^{2}-15yz^{2} \end{array}\right]\left[\begin{array}{c} m_{x}\\ m_{y}\\ m_{z} \end{array}\right],$$

where *x*, *y*, and *z* represent the three components of the distance vector *r*.

Nara [10] derived the formula for single-point magnetic gradient tensor localization of a magnetic target from the magnetic dipole magnetic field, as shown below:

(5) $$r=\left[\begin{array}{c} x\\ y\\ z \end{array}\right]=-3{\left[\begin{array}{ccc} \partial B_{x}/\partial x & \partial B_{x}/\partial y & \partial B_{x}/\partial z\\ \partial B_{y}/\partial x & \partial B_{y}/\partial y & \partial B_{y}/\partial z\\ \partial B_{z}/\partial x & \partial B_{z}/\partial y & \partial B_{z}/\partial z \end{array}\right]}^{-1}\left[\begin{array}{c} B_{x}\\ B_{y}\\ B_{z} \end{array}\right].$$

From Equation (5), it can be inferred that the real-time localization of a magnetic target can be achieved through the magnetic gradient tensor and the magnetic field three-component measurement information from a single measurement point. However, the mentioned single-point linear localization method faces a significant challenge in practical applications due to the substantial impact of geomagnetic field estimation errors on the localization results. Consequently, several scholars [14,19] have proposed methods utilizing two-point magnetic gradient tensors for localization, aiming to mitigate the influence of geomagnetic field estimation errors in practical applications. The general idea is to establish an optimization model that only includes measurements of magnetic gradient tensors and target positions. By using optimization algorithms, the method achieves the solution of target position parameters. This approach avoids the direct use of magnetic field components for positioning, thereby mitigating the impact of geomagnetic field estimation errors. Below is an introduction to a method proposed in this paper for establishing such an optimization model.

Transforming Equation (4), let us define vector Q and matrix K, with the expressions as follows: $Q=\left[\begin{array}{c} B_{xx}\\ B_{xy}\\ B_{xz}\\ B_{yy}\\ B_{yz} \end{array}\right],$ $K=\frac{\mu }{4\pi r^{7}}\left[\begin{array}{ccc} 9xr^{2}-15x^{3} & 3yr^{2}-15x^{2}y & 3zr^{2}-15x^{2}z\\ 3yr^{2}-15x^{2}y & 3xr^{2}-15xy^{2} & -15xyz\\ 3zr^{2}-15x^{2}z & -15xyz & 3xr^{2}-15xz^{2}\\ 3xr^{2}-15xy^{2} & 9yr^{2}-15y^{3} & 3zr^{2}-15y^{2}z\\ -15xyz & 3zr^{2}-15y^{2}z & 3yr^{2}-15yz^{2} \end{array}\right].$ From the expression, it can be concluded that Q is a vector dependent solely on the measured magnetic gradient tensor values, and K is a matrix dependent solely on the target position. Simplifying Equation (4), we obtain

(6) Q=KM.

Multiplying both sides of Equation (6) by the inverse matrix of ***K***, we obtain the following expression:

(7) $$K^{-1}Q=M.$$

The above is the expression for a single point. If we measure the magnetic gradient tensor information at two measurement points, the expression is as follows:

(8) $$\begin{array}{c} \left\{\begin{array}{c} K_{1}^{-1}Q_{1}=M\\ K_{2}^{-1}Q_{2}=M \end{array}\right. \end{array},$$

where the subscript 1 represents the relevant parameters for the first measurement point and subscript 2 represents the relevant parameters for the second measurement point. From Equation (8), we can obtain the following:

(9) $$K_{2}K_{1}^{-1}Q_{1}=Q_{2}.$$

According to Equation (9), based on the relative positional relationship between the two measurement points, the expression for measurement point 2 can be derived from measurement point 1. Based on Equation (9), we can establish the following objective function:

(10) $$f=\min{\left\|K_{2}K_{1}^{-1}Q_{1}-Q_{2}\right\|}_{2}.$$

In the above expression, matrix $K_{1}$ and $K_{2}$ are matrices solely dependent on the target position and are the quantities to be solved for, while $Q_{1}$ and $Q_{2}$ are the measured values of magnetic gradient tensors at two measurement points and are known quantities. By establishing an optimization model with Equation (10) as the objective function, the solution for the target position can be obtained through optimization algorithms. The optimization model described above does not include magnetic field vector terms; hence, it is not affected by geomagnetic field estimation errors. Research in [20] has shown that the optimization of the aforementioned objective function faces the challenge of potential convergence to false solutions, leading to a lower success rate and larger localization errors. Numerous studies suggest that introducing constraints into the objective function can more effectively address these issues [29].

### 2.2. Geometric Invariants

The magnetic gradient tensor exhibits many important invariant relationships, with invariants attracting widespread attention due to their property of remaining unchanged with variations in the target coordinate system. Among these invariants, geometric invariants find extensive application. Geometric invariants involve relationships between the distance vector, magnetic moment vector, and tensor eigenvectors. The proof for this is demonstrated as follows.

**Figure 1:**
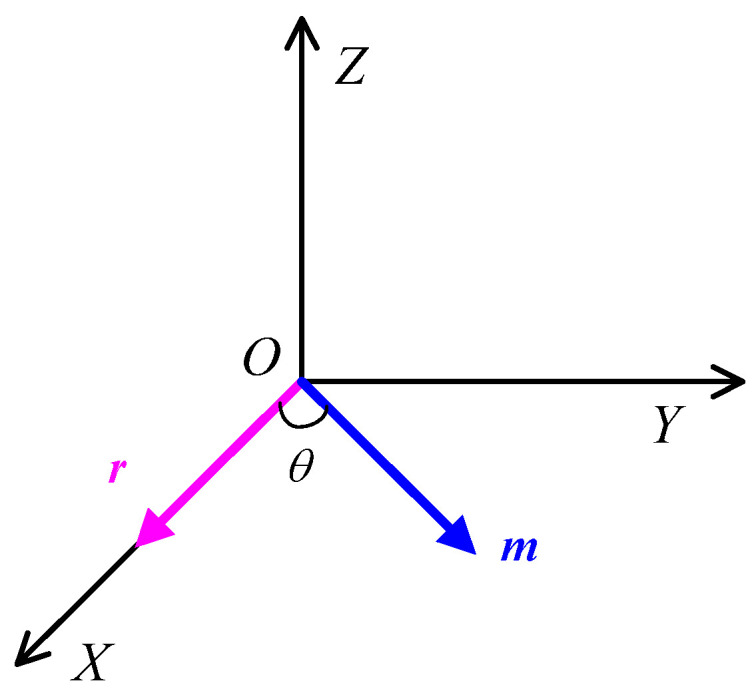
Geometric representation of the relationship between the distance vector and the target magnetic moment.

Begin by establishing a three-dimensional Cartesian coordinate system, as illustrated in Figure 1.

Assume that the magnetic target is located at the coordinate origin *O*, with the distance vector and target magnetic moment lying in the plane *XOY*, where the distance vector is represented by ***r*** = (*r*, 0, 0) and the target magnetic moment by ***m*** = (mcosθ,msinθ,0).Given the expressions for the distance vector and the target magnetic moment, substituting them into Equation (3) yields the expressions for the nine components of the magnetic gradient tensor. The expression for the magnetic gradient tensor [2] is as follows:

(11) $$G=\left[\begin{array}{ccc} -\frac{3\mu }{2\pi }\frac{m}{r^{4}}\cos\theta & \frac{3\mu }{4\pi }\frac{m}{r^{4}}\sin\theta & 0\\ \frac{3\mu }{4\pi }\frac{m}{r^{4}}\sin\theta & \frac{3\mu }{4\pi }\frac{m}{r^{4}}\cos\theta & 0\\ 0 & 0 & \frac{3\mu }{4\pi }\frac{m}{r^{4}}\cos\theta \end{array}\right].$$

Calculating Equation (11) yields the three eigenvalues of the magnetic gradient tensor, arranged in descending order as follows:

(12) $$\left\{\begin{array}{l} \lambda_{1}=\frac{3\mu m}{8\pi r^{4}}(-\cos\theta +\sqrt{4+5\cos^{2}\theta })\\ \lambda_{2}=\frac{3\mu m}{4\pi r^{4}}\cos\theta \\ \lambda_{3}=\frac{3\mu m}{8\pi r^{4}}(-\cos\theta -\sqrt{4+5\cos^{2}\theta }) \end{array}\right..$$

The corresponding eigenvectors for the three eigenvalues are as follows:

(13) $$\left\{\begin{array}{l} v_{1}=\left[\begin{array}{ccc} \frac{\sqrt{4+5\cos^{2}\theta }}{2\sin\theta }-\frac{3\cos\theta }{2\sin\theta } & 1 & 0 \end{array}\right]\\ v_{2}=\left[\begin{array}{ccc} 0 & 0 & 1 \end{array}\right]\\ v_{3}=\left[\begin{array}{ccc} -\frac{\sqrt{4+5\cos^{2}\theta }}{2\sin\theta }-\frac{3\cos\theta }{2\sin\theta } & 1 & 0 \end{array}\right] \end{array}\right..$$

From the expression of the eigenvectors, it can be deduced that eigenvectors ***v***_1_ and ***v***_3_ lie in the plane *XOY*, co-planar with the distance vector ***r*** and the target magnetic moment ***m***. Eigenvector ***v***_2_ is perpendicular to this plane, hence orthogonal to the distance vector ***r*** and the target magnetic moment ***m***. Exploiting this property enables the precise localization of the target. Therefore, the relationship satisfied by the eigenvector corresponding to the smallest absolute eigenvalue [2] is as follows:

(14) $$\left\{\begin{array}{c} v_{2,1}\cdot r_{1}=0\\ v_{2,2}\cdot r_{2}=v_{2,2}\cdot (r_{1}+\Delta r)=0 \end{array}\right.,$$

where the second digit indexed in both “***v***_2,1_” and “***v***_2,2_” corresponds to the measurement point. The first digit represents the order of arranging the eigenvalues in descending order, and subscript “2” represents the eigenvector corresponding to the second eigenvalue, which is the eigenvector perpendicular to the distance vector and the magnetic moment. Adding the constraint of the eigenvector to Equation (10) and transforming it yields the following expression:

(15) $$\begin{array}{c} 1\\ V=\left[\begin{array}{c} K_{2}K_{1}^{-1}Q_{1}-Q_{2}\\ v_{2,1}\cdot r_{1}\\ v_{2,2}\cdot (r_{1}+\Delta r) \end{array}\right]\begin{array}{c} \;5\\ \;1\\ \;1 \end{array} \end{array}$$

(16) $$f=\min{\left\|V\right\|}_{2},$$

where ***V*** represents the 7 × 1 vector containing the target position (*x*, *y*, *z*). Solving Equation (16) allows for the determination of the target’s position, achieving localization. Equation (16) constitutes a typical nonlinear optimization problem. The solution methods for the above problem can be divided into two categories: traditional methods, such as Newton’s iteration method, the conjugate gradient method, the LM algorithm, etc.; and intelligent optimization algorithms, such as the genetic algorithm, the particle swarm optimization algorithm, etc. Intelligent optimization algorithms can increase computational accuracy by increasing the size of the population and the number of iterations, but this comes with an increase in computation time. In target localization, both computation time and localization accuracy are crucial. Therefore, this paper utilizes the LM algorithm for optimal solution computation. The LM algorithm combines the advantages of gradient descent and Newton’s method, but it requires higher precision in the initial values.

### 2.3. The Levenberg–Marquardt Algorithm

The Levenberg–Marquardt (LM) algorithm [30,31] is a typical method for solving nonlinear optimization problems, but it exhibits sensitivity to the initial values. In addressing the mentioned issue, this paper proposes using the results of the Nara single-point localization method as the initial values for the LM algorithm. Subsequently, the LM algorithm is employed to obtain an accurate solution for two-point localization.

The LM algorithm is an iterative method that aims to minimize the objective function through repeated iterations, ultimately determining the optimal solution. The fundamental principle of the LM algorithm involves a first-order Taylor expansion for a nonlinear function *f*

(17) f(x+Δx)≈f(x)+J(x)Δx,

where ***J***(***x***) is the Jacobian matrix, representing the derivative of *f*(***x***) with respect to its variables ***x***. The LM algorithm aims to find a parameter vector Δx that minimizes ${\left\|f(x+\Delta x)\right\|}^{2}$. In other words, it seeks a solution to the following problem:

(18) $$\underset{\Delta x}{\min}\frac{1}{2}{\left\|f(x)+J(x)\Delta x\right\|}^{2}.$$

The idea of the Gaussian–Newton method is to expand the above expression and differentiate it with respect to Δx, resulting in

(19) $$\Delta x=-{\left[J{\left(x\right)}^{T}J(x)\right]}^{-1}J{\left(x\right)}^{T}f(x).$$

In practical solving, the Hessian matrix $H(x)=J{\left(x\right)}^{T}J(x)$ in the above expression may be approximately singular, making inversion impossible. To address this issue, the LM algorithm introduces a damping factor μ(μ>0) on top of the Gaussian–Newton method.

(20) $$\Delta x=-{\left[J{\left(x\right)}^{T}J(x)+\mu I\right]}^{-1}J{\left(x\right)}^{T}f(x),$$

where the damping factor μ ensures the invertibility of $J{\left(x\right)}^{T}J(x)+\mu I$, enhancing the algorithm’s robustness. The value of the damping factor is obtained empirically and adjusted based on the observed descent effectiveness. The expression for descent effectiveness is given by

(21) $$\rho =\frac{{\left\|f(x)\right\|}_{2}^{2}-{\left\|f(x)+\Delta x\right\|}_{2}^{2}}{{\left\|J(x)\Delta x\right\|}_{2}^{2}},$$

where the numerator represents the actual descent value, while the denominator corresponds to the descent value under the approximate model. When ρ approaches 1, it indicates a suitable value; when ρ is too small, it suggests a suboptimal descent, and μ needs to decrease. If ρ is relatively large, it signifies a greater-than-expected descent, requiring an increase in μ. When μ≈0, the term $J{\left(x\right)}^{T}J(x)$ dominates, and the LM algorithm approaches the Gaussian–Newton method. As μ increases, μI takes precedence, making the LM algorithm more akin to gradient descent. The precise solution for the target’s position can be iteratively determined based on Equation (20).

## 3. Simulations

**Figure 2:**
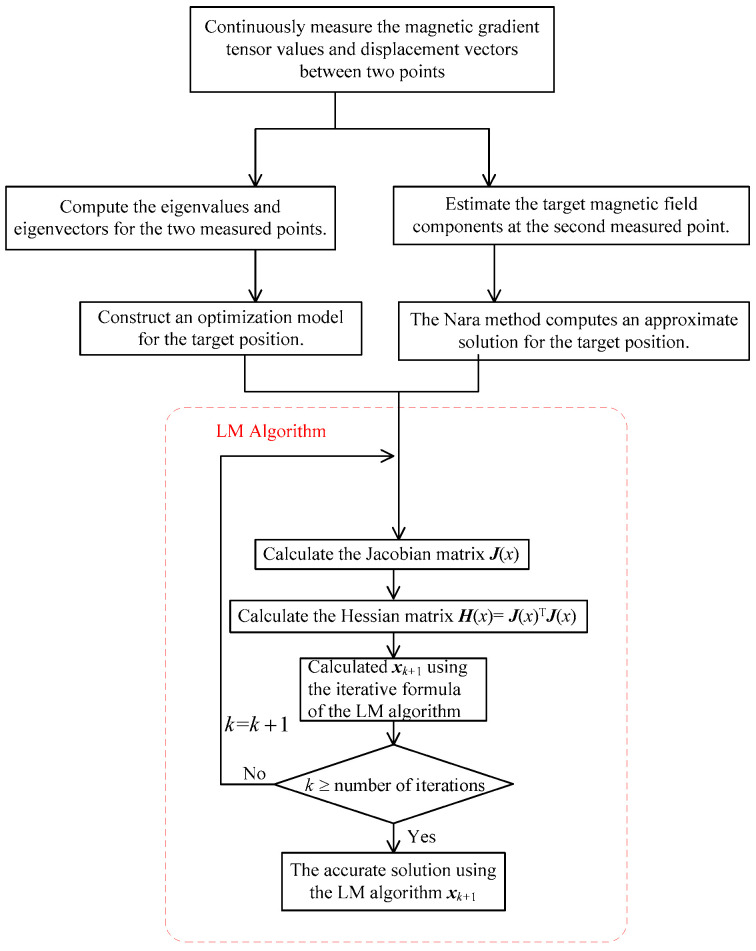
The flowchart of the positioning algorithm proposed in this paper.

To validate the effectiveness of the method proposed in this paper, simulation experiments were designed for verification. The computational process used in the simulation experiments of the method proposed in this paper is illustrated in Figure 2. For comparison with existing methods, the Nara localization method, a two-point localization method from reference [19], and another two-point localization method from reference [20] were selected. For simplicity, the localization method from reference [19] is referred to as the ZTPT (Zhang’s two-point tensor) method, the localization method from reference [20] is referred to as the LTPT (Liu’s two-point tensor) method, and the proposed localization method in this paper is referred to as the PTPT (the proposed two-point tensor) method.

**Figure 3:**
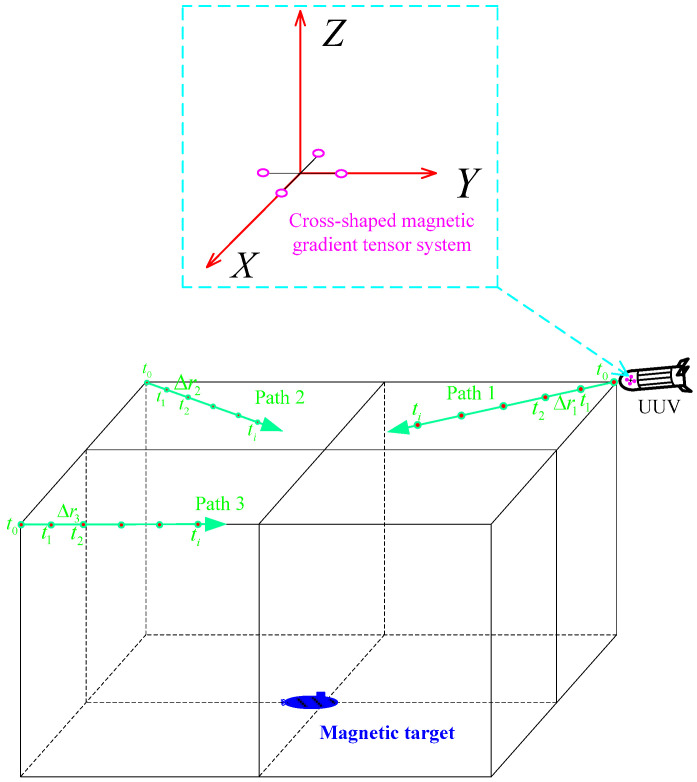
Simulation-based positioning experiment.

The simulation conditions are as follows: the magnetic gradient tensor measurement system is mounted on an underwater UUV platform for magnetic target localization. The commonly seen structural forms of magnetic gradient tensor systems include cross-shaped, square, tetrahedral, and hexahedral forms. Among them, the cross-shaped magnetic gradient tensor system is relatively easy to implement, and the center point of the structure is easy to determine. According to reference [32], the system error of the cross-shaped magnetic gradient tensor system is relatively small. Therefore, in simulation experiments, the cross-shaped magnetic gradient tensor system is used for target localization. To minimize magnetic interference from the UUV platform to the detection system, the system is installed at the head of the UUV, as shown in Figure 3. A Cartesian coordinate system is established with the center of the cross-shaped magnetic gradient tensor system as the origin. The baseline length of the system (the distance between two magnetic sensors on the same coordinate axis) is 0.5 m. The three measurement axes of the fluxgate magnetometer are overlaid with Gaussian white noise with a variance of 0.06 nT. The magnetic moment vector of the magnetic target is (4,000,000, 200,000, 100,000) Am^2^. The geomagnetic field during simulation is simulated using the WMM2020 geomagnetic model. The model’s input latitude and longitude are 36°06′ N, 120°19′ E, and the time is January 2023.

The factors that may affect PTPT localization mainly include different initial values, different measurement routes, different measurement noise conditions, and different UUV platform position measurement errors. Therefore, simulation experiments are separately designed to analyze these factors. To verify the localization effectiveness of PTPT under different measurement paths, three typical measurement paths are selected for simulation, as shown in Figure 3. Under the path 1 condition, at time t_0_, the magnetic target is located at coordinates (40, −40, −40) m, and the displacement between two adjacent measurement points, $\Delta r_{1}$, is (4, −4, 0) m. Under the path 2 condition, at time t_0_, the magnetic target is located at coordinates (40, 40, −40) m, and the displacement between two adjacent measurement points, $\Delta r_{2}$, is (4, 4, 0) m. Under the path 3 condition, at time t_0_, the magnetic target is located at coordinates (−40, 40, −40) m, and the displacement between two adjacent measurement points, $\Delta r_{3}$, is (0, 4, 0) m. During simulation, according to the algorithm process in this paper, as shown in Figure 2, the target position at time t_2_ is calculated based on the measurement data of two consecutive measurement points, t_1_ and t_2_. The localization error E and the relative localization error ε are calculated based on the calculated target position and the actual target position using the following equations:

(22) $$\left\{\begin{array}{l} E={\left\|r_{e}-r_{t}\right\|}_{2}\\ \varepsilon =\frac{{\left\|r_{e}-r_{t}\right\|}_{2}}{{\left\|r_{t}\right\|}_{2}} \end{array}\right.,$$

where $r_{t}=(x_{t},y_{t},z_{t})$ represents the true value of the target’s position, and $r_{e}=(x_{e},y_{e},z_{e})$ is the estimated target position obtained through the localization algorithm. Therefore, the localization error E and the relative localization error ε are calculated based on the comparison between the true and estimated positions.

### 3.1. Analysis of Positioning Results under Different Initial Conditions

Under different initial value conditions, the localization results of the PTPT method are analyzed. In the PTPT method, the LM algorithm is used to solve the nonlinear equations. However, the LM algorithm has some dependence on initial values, and inappropriate initial values may lead to failure in finding the optimal solution. To illustrate this situation, different initial values have been selected for solving the equation using the LM algorithm. Taking path 1 from Figure 3 as an example for analysis, a coordinate system is established with the center of the measurement system as the origin. At the initial time t_0_, the magnetic target is located at coordinates (40, −40, −40) m. At time t_1_, the displacement of the UUV forward movement is (4, −4, 0) m. At this time, in the coordinate system with the center of the measurement system as the origin, the magnetic target is located at position (36, −36, −40) m. Utilizing the magnetic gradient tensor measurement data at t_0_ and t_1_, along with the position relationship between the two points, the target position can be solved. Table 1 presents the results obtained by the LM algorithm under different initial value conditions.

**Figure 4:**
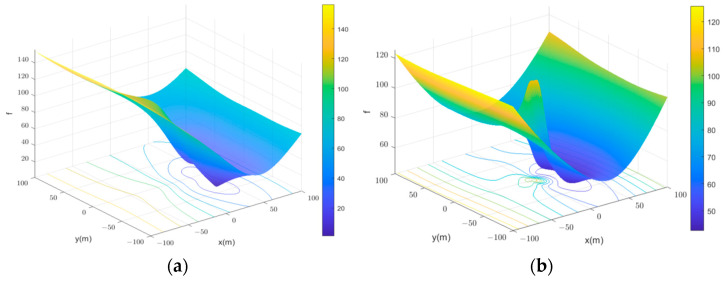
The distribution of the objective function values for points on different planes: (**a**) Z = −40 m; (**b**) Z = −10 m.

In Table 1, the first column represents the different initial values of the LM algorithm, the second column represents the target positions obtained by the LM algorithm, and the third column represents the relative error. From the simulation results, it can be observed that the LM algorithm can achieve optimal solutions when the initial values fall within a certain range of the true target position. If the initial values deviate beyond a specific range from the true target’s position, the LM algorithm may fail to converge to an optimal solution. Different planes have been selected to calculate the objective function values for points on those planes. Two typical planes, Z = −40 m and Z = −10 m, have been selected. The simulation results are shown in Figure 4. From the simulation graph, it can be observed that the objective function distribution on the Z = −10 m plane exhibits multiple local minima, making it prone to getting trapped in local optima. On the Z = −40 m plane, there are two local minima, with one representing the true target position as the global minimum point. If the initial values are chosen closer to the global minimum point, the LM algorithm can compute the global minimum point. In practical scenarios, despite the Nara localization method being influenced by geomagnetic field estimation errors and having some inherent inaccuracies, it provides an accurate solution within a certain range of the true target position. Therefore, using the target position calculated by the Nara method as an initial value in the LM algorithm can yield a precise solution for the target position.

### 3.2. Analysis of Path 1 Positioning Results

**Figure 5:**
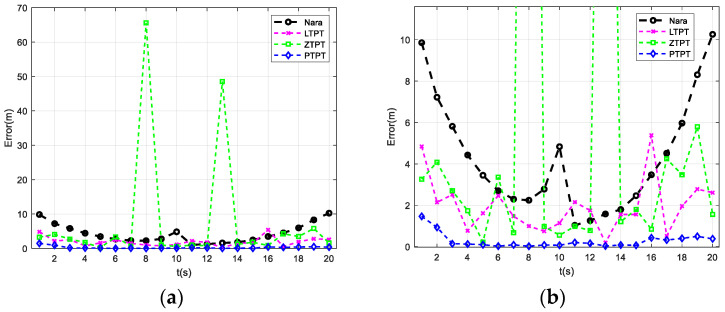
Localization results when the platform moves along path 1: (**a**) localization results of different algorithms; and (**b**) results with a local zoom display along the y-axis.

The positioning results are computed using the Nara, ZTPT, LTPT, and PTPT methods. Figure 5 illustrates the calculation results. Figure 5a displays the variation in the positioning errors over time for the four methods. However, due to the presence of two significant error peaks in the results of the ZTPT method, it is challenging to visually discern the differences between the four positioning methods in Figure 5a. Therefore, to enhance clarity, Figure 5b zooms in on the y∈[0,12] of Figure 5a.

From Figure 5b, it is evident that, among the four positioning methods, the PTPT method proposed in this paper consistently exhibits smaller positioning errors, indicating the best positioning performance. Due to the presence of geomagnetic field estimation errors, the Nara method exhibits relatively larger positioning errors. The ZTPT method, although not directly utilizing magnetic field tri-components for positioning, exhibits slightly smaller errors compared to the Nara method. However, it faces the issue of easily falling into local optimal solutions, as evidenced by the high calculation errors at t = 8 s and t = 13 s, reaching 65.6 m and 48.5 m, respectively, as shown in Figure 5a. The LTPT method incorporates the constraint terms, effectively resolving the issues present in the ZTPT method. Nevertheless, as geometric invariance constraints are not included, significant errors still persist. With the addition of vertical vector constraints, the PTPT method further reduces the positioning errors. The statistical analysis of the positioning errors along path 1 reveals that the PTPT method has an average positioning error of 0.28 m, the lowest among the four methods. The Nara method has an average positioning error of 4.32 m, while the LTPT method’s average positioning error is 1.96 m. Due to the presence of two significant error peaks, the average positioning error of the ZTPT method is 7.62 m. However, upon excluding these two large error points, the average positioning error reduces to 2.13 m, still lower than that of the Nara method.

### 3.3. Analysis of Path 2 Positioning Results

**Figure 6:**
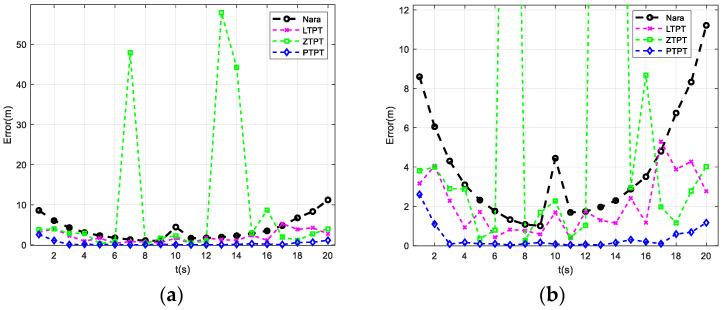
Localization results when the platform moves along path 2: (**a**) localization results of different algorithms; and (**b**) results with a local zoom display along the y-axis.

The simulation analysis of the UUV’s positioning performance along path 2 using the four methods is presented in Figure 6. Figure 6a shows the original processing results, while Figure 6b zooms in on the y-region of Figure 6a. From the simulation results, it is evident that the positioning error of the PTPT method proposed in this paper consistently outperforms the other three methods. The ZTPT method still exhibits susceptibility to falling into local optimal solutions, with significant positioning errors occurring at t = 7 s, t = 13 s, and t = 14 s, reaching 47.89 m, 57.87 m, and 44.25 m, respectively. When a magnetic moment constraint is added to the ZTPT method, the positioning error of the LTPT method significantly decreases. However, the problem of susceptibility to local optimal solutions persists, with a positioning error of 5.3 m at t = 28 s, exceeding the 4.8 m positioning error of the Nara method. The statistical analysis of the average positioning errors of the four methods reveals that the Nara method has an average positioning error of 3.95 m, while the LTPT method’s average positioning error is 2.04 m, lower than that of the Nara method. The average positioning error of the ZTPT method is 9.6 m, but, upon removing the three points with significant errors, the average positioning error reduces to 2.47 m. The positioning error of the PTPT method is 0.39 m, indicating the best positioning performance.

### 3.4. Analysis of Path 3 Positioning Results

**Figure 7:**
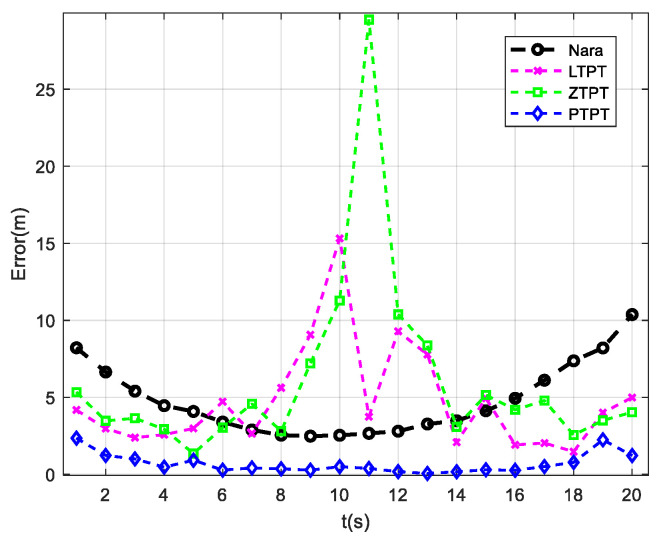
Localization results when the platform moves along path 3.

The simulation analysis of the positioning effect of UUV along path 3 motion isconducted using the Nara method, the LTPT method, the ZTPT method, and the PTPT method to locate the target. The positioning errors of different methods are calculated, as shown in Figure 7, and it can be observed that both the LTPT method and the ZTPT method suffer from the problem of getting trapped in local optimal solutions. The positioning error of the LTPT method at t = 10 s reaches 15.32 m, while the ZTPT method’s positioning error at t = 11 s is 29.52 m. The statistical analysis of the average positioning errors for several methods reveals that the Nara method has an average positioning error of 4.8 m. Due to points with significant positioning errors, the ZTPT method has an average positioning error of 6.06 m, higher than that of the Nara method. The LTPT method has an average positioning error of 4.74 m, slightly lower than that of the Nara method. The PTPT method proposed in this paper shows the best positioning effect, with a positioning error of only 0.7 m, the lowest among all the methods.

### 3.5. Analysis of Positioning Results under Different Sensor Noise Conditions

**Figure 8:**
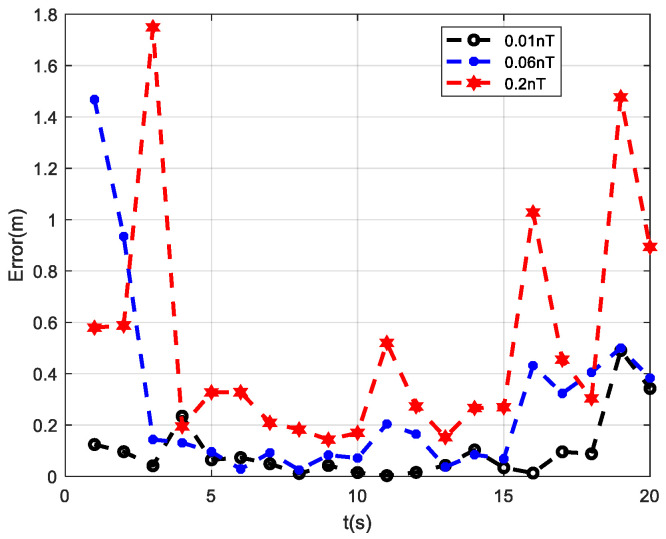
Positioning results under different sensor noise conditions.

Based on the simulation results of the above three typical measurement paths, it can be concluded that, among the four positioning methods, the PTPT method exhibits the smallest positioning error and the best performance. To validate the positioning effectiveness of the PTPT method under different sensor noise conditions, the following simulation experiment was designed. Path 1 was used for the simulation, and different measurement noises were added to the three measurement axes of the fluxgate sensors in the measurement system. The variances in the noises were set to 0.01 nT, 0.06 nT, and 0.2 nT, respectively. Then, the PTPT method proposed in this paper was employed to locate the target, and the calculation results are shown in Figure 8.

From the simulation results, it can be observed that, when the three measurement axes of the fluxgate sensors are subjected to noise with a variance of 0.01 nT, the average positioning error of the PTPT method is 0.1 m. When the noise variance is increased to 0.06 nT, the average positioning error becomes 0.28 m, and, when it reaches 0.2 nT, the average positioning error further increases to 0.51 m. As the measurement noise increases, the average positioning error of the PTPT method continues to rise. However, even when the measurement noise is 0.2 nT, the average positioning error remains below 1 m. Therefore, the PTPT method proposed in this paper demonstrates insensitivity to measurement noise and exhibits good robustness.

**Figure 9:**
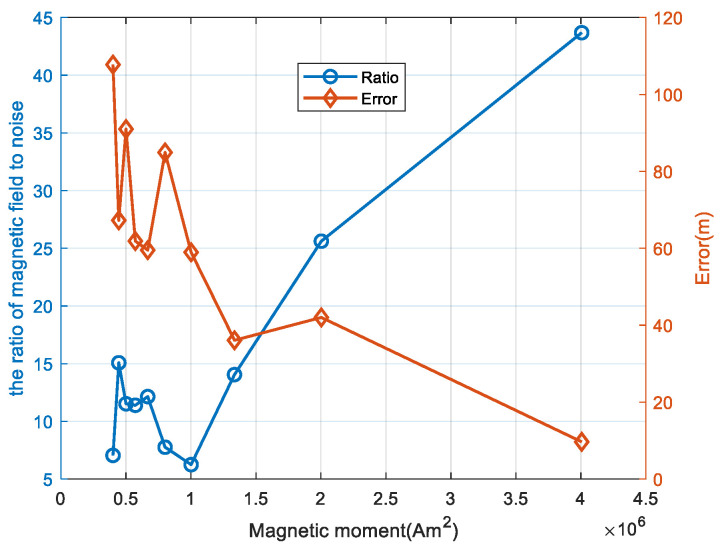
Relationship between target positioning error and target magnetic moment variation.

The robustness of the PTPT method may not be suitable for all levels of noise intensity, which is speculated to be related to the size of the target magnetic moment and the level of noise. A simulation was conducted to examine the effect of target magnetic moment variations on localization errors when the noise variance in the magnetic fluxgate was 1 nT. During simulation, the target magnetic moment values were varied from the original value to one-tenth of the original value, with the intermediate values being half, one-third, and so forth. The simulation scenario involved a UUV platform moving along path 1, and localization of the target was performed using measurement data from two points, t0 and t1. The relationship between the calculated localization error and the target magnetic moment is shown in Figure 9. Additionally, the relationship between the ratio of the target’s magnetic anomaly signal magnitude to the measurement noise magnitude and the target’s magnetic moment variations was analyzed, as depicted in Figure 9. In Figure 9, the light red curve represents the relationship between the target’s localization error and its magnetic moment variations, with the corresponding error values displayed on the right axis. The blue curve represents the relationship between the ratio and the target’s magnetic moment variations, with the corresponding ratio values displayed on the left axis.

From the simulation results in Figure 9, it can be observed that, under constant noise levels, as the magnetic moment decreases, the target positioning error increases continuously. When the ratio of the target’s magnetic anomaly magnitude to the measurement noise magnitude is less than 15, corresponding to a magnetic moment of less than 1,335,420 Am², the target positioning error increases rapidly.

### 3.6. Analysis of the Influence of Position Measurement Errors on the Localization Results for a UUV Platform

**Figure 10:**
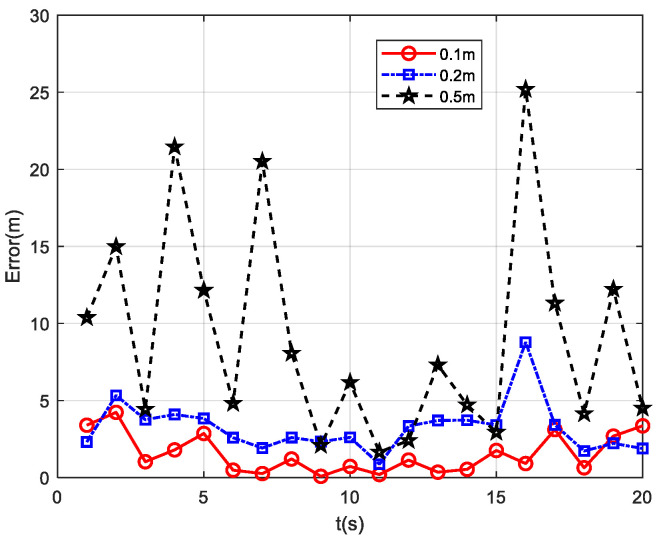
Relationship between UUV platform’s position measurement error and localization error.

The above simulation analysis assumes that the position of the magnetic sensor, i.e., the UUV carrier platform’s position, is accurate. However, in reality, the UUV platform itself has position measurement errors. If there are errors in the position of the magnetic sensor, it will affect the localization performance of the PTPT method proposed in this paper. In a simulation analysis where the variances in the UUV platform’s self-position measurement errors are 0.1 m, 0.2 m, and 0.5 m, respectively, the localization performance of the PTPT method is evaluated. Path 2 is chosen for the simulation experiments, and the simulation results are shown in Figure 10.

From the simulation results, it can be observed that, as the distance between the UUV platform and the magnetic target decreases, the localization error of the magnetic target decreases continuously. Moreover, the larger the position measurement error of the UUV platform, the relatively larger the localization error of the magnetic target. When the variance in the position measurement error is 0.1 m, the average localization error is minimized, measuring 1.54 m. When the variance in the position measurement error is 0.2 m, the average error is 3.22 m. Lastly, when the variance in the position measurement error is 0.5 m, the maximum average localization error is 9.07 m.

### 3.7. Analysis of the Localization Effects of Different Objective Functions

**Figure 11:**
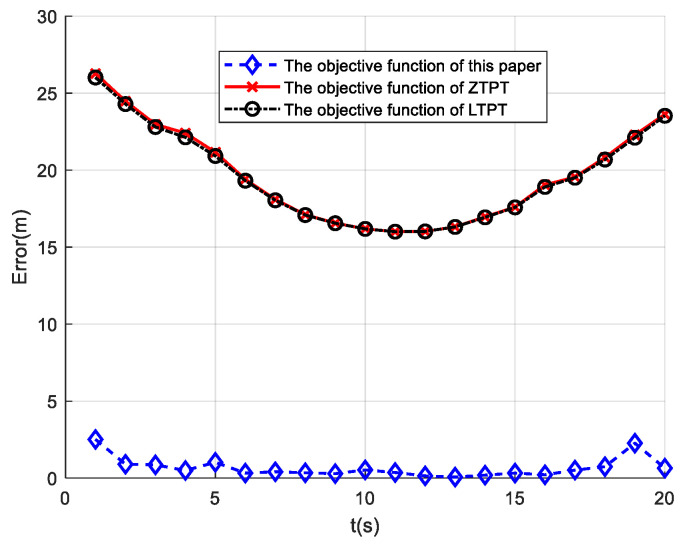
Comparison of localization effects under different objective functions.

The objective function proposed in this paper’s PTPT method incorporates constraints on geometric invariants. To verify the localization effects under different objective functions, this study compares the objective functions used in the LTPT method and the ZTPT method. When simulating the comparisons, only the objective functions are altered, while the rest of the conditions are set to the same values as the PTPT method. The initial values are computed using the Nara method, and Path 3 is selected for the simulation. The simulation results are depicted in Figure 11 below.

From the simulation results, it can be observed that the objective function incorporating geometric invariants achieves the best localization effect, with an average localization error of 0.66 m. In contrast, the localization effects of the objective functions used in the LPTP method and ZTPT method are poorer, with average localization errors of 19.55 m and 19.64 m, respectively. It is worth noting that the LPTP method adds a magnetic moment constraint to the objective function of the ZTPT method, resulting in a slightly lower localization error compared to the ZTPT method.

## 4. Experiments and Result Analysis

**Figure 12:**
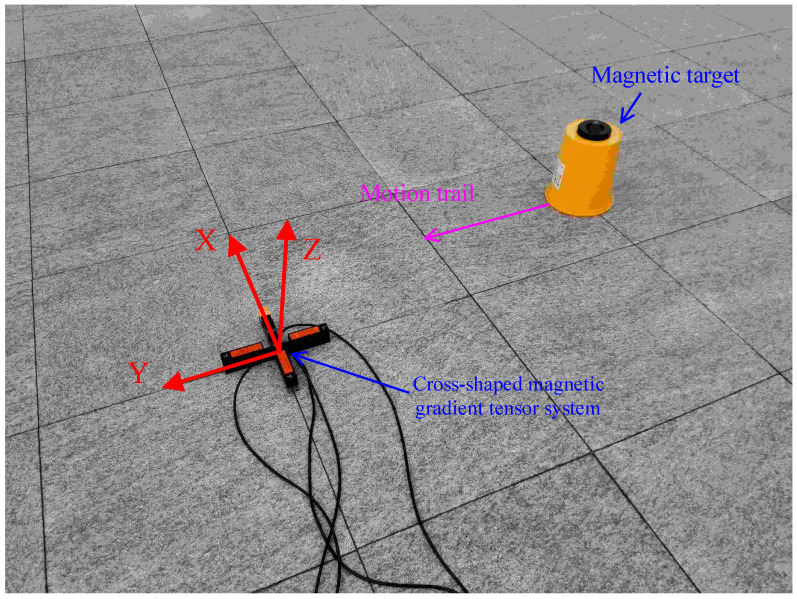
Localization method validation experiment.

To further validate the effectiveness and practicality of the proposed method, this study conducted real positioning experiments with ferromagnetic objects. The experiment took place in a plaza with minimal environmental magnetic field interference, as depicted in Figure 12. A cross-shaped magnetic gradient tensor system was constructed using four domestically produced magnetic fluxgate magnetometers. The stand was made of non-magnetic materials, and the system’s baseline length was 0.26 m. The system errors were calibrated before use. During the experiment, a coordinate system was established with the center of the cross-shaped magnetic gradient tensor system as the origin, as shown in Figure 12.

A ferromagnetic object was placed on a plastic bucket during the experiment, with the bucket’s height being 0.25 m. The magnetic gradient tensor system remained fixed, and the magnetic target was moved parallel to the Y-axis to simulate multi-point measurements. The magnetic target started at y = −1.2 m and then was moved to y = 1.2 m in increments of 0.3 m, resulting in a total of nine measurement points. The data from these points were processed using four different methods: Nara, PTPT, LTPT, and ZTPT.

**Figure 13:**
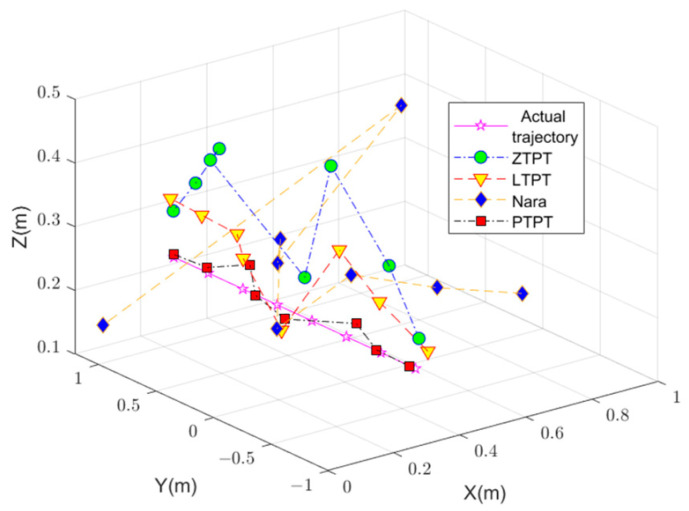
Spatial distribution of the actual position of the target and the calculated positions by different algorithms.

**Figure 14:**
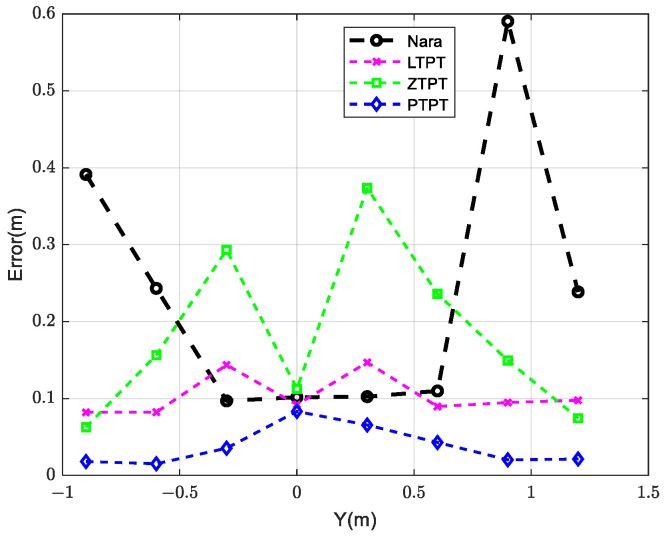
Comparison of positioning errors of different algorithms.

The positioning results, shown in Figure 13 and Figure 14 and Table 2, reveal that PTPT provides the most accurate positioning. The spatial distribution of the calculated positions closely aligns with the actual target positions. Figure 13 illustrates the quantitative comparison of the positioning errors among the different algorithms. Due to estimation errors in the geomagnetic field, Nara’s method exhibits larger positioning errors when the measurement system is far from the magnetic target but decreases when closer. PTPT, LTPT, and ZTPT overcome the impact of geomagnetic field estimation errors, resulting in relatively smaller positioning errors. However, ZTPT faces the challenge of getting stuck in local optimal solutions, as seen in specific positions. LTPT, although slightly improving on ZTPT’s positioning errors, still has the potential to fall into local optimal solutions. The proposed PTPT method consistently demonstrates smaller positioning errors, making it capable of achieving a global optimal solution.

A statistical analysis of the relative errors calculated by the different positioning methods is presented in Table 2. The table provides the average errors for the X, Y, and Z directions as well as the magnitude of the position vector. The results indicate that Nara’s method yields the highest average error in the X direction at 64.6%, while PTPT exhibits the lowest at 9.4%. For the Y direction, Nara’s method has the smallest average error at 11.1%, and ZTPT has the largest at 84.4%. In the Z direction, PTPT has the lowest average error at 6.9%, and ZTPT has the highest at 70.7%. Due to the local optimal solutions in ZTPT’s results, the average error is relatively high. Regarding the magnitude of the position vector, PTPT has the smallest average error at 7.3%, and ZTPT has the largest at 32.2%. This experimental verification effectively demonstrates the validity of the method proposed in this paper.

## 5. Conclusions

To address the challenges of significant estimation errors in geomagnetic fields and the tendency to fall into local optimal solutions in current magnetic gradient tensor-based positioning methods, a two-point positioning method based on tensor geometric invariants is proposed. This method initially establishes an optimization model for the target’s position by combining magnetic gradient tensor measurements and geometric invariant constraints. Subsequently, the Nara positioning method’s results are utilized as the initial values input into the LM algorithm to achieve an accurate solution for the target position.

This study compares the positioning performance of various common target localization methods (such as the Nara method, the LTPT method, and the ZTPT method) with the proposed method through both simulation experiments and real measurements. The results demonstrate that the proposed method can overcome the impact of geomagnetic field estimation errors, achieving precise target localization. In practical applications, the proposed positioning method can be integrated into a moving platform to measure the magnetic gradient tensor values of two points through the platform’s motion, enabling accurate target localization. Alternatively, it can be employed to construct a system capable of simultaneously measuring the magnetic gradient tensor information of two points for real-time target positioning.

## Figures and Tables

**Table 1:** The computational performance of the LM algorithm under different initial conditions.

The Initial Values	(*x_e_*, *y_e_*, *z_e_*)/m	(*ε_x_*_,_ *ε_y_*_,_ *ε_z_*)/%
(48.03, −45.67, −45.51)	(34.63, −35.06, −38.66)	(3.81, 2.61, 3.35)
(14.92, −77.19, −77.35)	(−12.09, −44.99, −46.62)	(133.58, 24.97, 16.55)
(20.05, −21.02, −19.05)	(34.63, −35.06, −38.66)	(3.81, 2.61, 3.35)
(0.71, 0.55, 0.65)	(0.84, 2.65, 1.68)	(97.67, 107.36, 104.20)
(1.25, 0.25, −0.02)	(116.52, 291.02, 294.63)	(223.67, 908.39, 836.58)
(75.58, −74.67, −74.89)	(34.63, −35.06, −38.66)	(3.81, 2.61, 3.35)
(−12.59, 14.19, 12.75)	(−155.48, 156.23, 161.22)	(531.89, 533.97, 503.05)

**Table 2:** Table of relative errors calculated by different positioning methods.

	Relative Localization Error	*ε_x_* (%)	*ε_y_* (%)	*ε_z_* (%)	*ε_r_* (%)
Method					
ZTPT		16.2	84.4	70.7	32.2
LTPT		12.3	19.4	34.9	17.2
Nara		64.6	11.1	36.9	29.9
PTPT		9.4	17.8	6.9	7.3

## Data Availability

The data that support the findings of this study are available from the corresponding author upon reasonable request.
